# Tuberculous Meningitis in Children: A Forgotten Public Health Emergency

**DOI:** 10.3389/fneur.2022.751133

**Published:** 2022-03-17

**Authors:** Karen du Preez, Helen E. Jenkins, Peter R. Donald, Regan S. Solomons, Stephen M. Graham, H. Simon Schaaf, Jeffrey R. Starke, Anneke C. Hesseling, James A. Seddon

**Affiliations:** ^1^Desmond Tutu TB Centre, Department of Paediatrics and Child Health, Faculty of Medicine and Health Sciences, Stellenbosch University, Cape Town, South Africa; ^2^Department of Biostatistics, School of Public Health, Boston University, Boston, MA, United States; ^3^Department of Paediatrics and Child Health, Faculty of Medicine and Health Sciences, Stellenbosch University, Cape Town, South Africa; ^4^Department of Paediatrics and Murdoch Children's Research Institute, Centre for International Child Health, University of Melbourne Royal Children's Hospital, Melbourne, VIC, Australia; ^5^International Union Against Tuberculosis and Lung Disease, Paris, France; ^6^Department of Pediatrics, Baylor College of Medicine, Houston, TX, United States; ^7^Department of Infectious Diseases, Imperial College London, London, United Kingdom

**Keywords:** tuberculosis, tuberculous meningitis, pediatric, children, surveillance

## Abstract

Tuberculous meningitis (TBM) remains a major cause of morbidity and mortality in children with tuberculosis (TB), yet there are currently no estimates of the global burden of pediatric TBM. Due to frequent non-specific clinical presentation and limited and inadequate diagnostic tests, children with TBM are often diagnosed late or die undiagnosed. Even when diagnosed and treated, 20% of children with TBM die. Of survivors, the majority have substantial neurological disability with significant negative impact on children and their families. Surveillance data on this devastating form of TB can help to quantify the contribution of TBM to the overall burden, morbidity and mortality of TB in children and the epidemiology of TB more broadly. Pediatric TBM usually occurs shortly after primary infection with *Mycobacterium tuberculosis* and reflects ongoing TB transmission to children. In this article we explain the public health importance of pediatric TBM, discuss the epidemiology within the context of overall TB control and health system functioning and the limitations of current surveillance strategies. We provide a clear rationale for the benefit of improved surveillance of pediatric TBM using a TB care cascade framework to support monitoring and evaluation of pediatric TB, and TB control more broadly. Considering the public health implications of a diagnosis of TBM in children, we provide recommendations to strengthen pediatric TBM surveillance and outline how improved surveillance can help us identify opportunities for prevention, earlier diagnosis and improved care to minimize the impact of TBM on children globally.

## Introduction

Tuberculous meningitis (TBM) is the most devastating form of tuberculosis (TB) in children, with high mortality and morbidity (1, 2). The neurological disability due to TBM has significant long-term consequences for children, their families and healthcare systems (3); prevention and early diagnosis are therefore critical. For programmes to effectively respond to this public health emergency, improved surveillance data are required to better define the burden of TBM in children and to inform interventional research (4). We address this critical need for improved surveillance, review current TB surveillance strategies and their limitations, and present practical guidelines for strengthening global pediatric TBM surveillance.

## Why Is Pediatric TBM a Public Health Priority?

Children (<15 years old) account for 12% of the estimated global TB burden (5). Young children (<5 years) or those living with HIV are at high risk for TB and TB-related mortality (6, 7). Despite availability of effective treatment, an estimated 1.19 million children globally developed TB in 2019 and 230,000 children died, most <5 years old, making TB a top ten cause of under-5 mortality (5, 8).

Children aged <2 years are at particularly high risk of disseminated TB including TBM (6). TBM frequently presents with non-specific symptoms during the early stages of disease, resulting in frequent delayed diagnosis and advanced clinical disease at eventual presentation. Although early diagnosis and treatment are key to reducing mortality and improving functional outcomes (9, 10), a systematic review found that nearly 50% of children with TBM were diagnosed at an advanced disease stage (2). Without treatment, TBM is fatal (1); undiagnosed cases, or those diagnosed late, likely make a substantial but unquantified contribution to global pediatric TB deaths.

Furthermore, TBM in children is preventable. First, Bacille Calmette-Guérin (BCG) vaccination at birth remains an effective intervention in high TB-burden countries to prevent TBM in young children (pooled efficacy of 73% (95% CI: 67–79%), (11). Second, TB transmission often occurs amongst family members and contact tracing combined with prompt provision of TB preventive therapy (TPT) following exposure to *Mycobacterium tuberculosis (M.tb)* also reduces the risk of developing TB, including TBM (12, 13). TPT is safe, effective (12), widely available and universally recommended for high-risk child contacts after *M.tb* exposure. However, implementation in high TB-burden settings remains poor (14), with TPT initiated in only one third of the estimated 1.3 million eligible child TB contacts in 2019 (5).

Therefore, the resulting mortality and morbidity in pediatric TBM often reflects health system failures. Every TBM diagnosis should be considered an opportunity to evaluate the health system and assessment of every child with TBM should be a standard quality control activity for TB programmes.

## The Epidemiology of TBM in Children – What Do We Know?

### TBM Pathogenesis and Clinical Features

The pathogenesis of pediatric TB and TBM is well-described. Following pulmonary infection with *M.tb*, a localized, pneumonic inflammatory process results in a parenchymal (Ghon) focus from which lympho-haematogenous dissemination of bacilli occurs throughout the body, establishing meningeal, and sometimes choroid plexus or ventricular wall foci (the Rich foci) (15). The contents of these foci may be discharged into the subarachnoid space 6–8 weeks later initiating a host inflammatory response with peri-vascular inflammation and basal exudates with resultant infarcts, cranial nerve palsies, and hydrocephalus (15). Bacillary dissemination is particularly common in infants (<1 year) and in young children (16, 17).

### Risk of Developing TB and TBM in Childhood

Young children are particularly prone to systemic *M.tb* dissemination post-infection. A review of pre-chemotherapy studies found that infants had a 20% risk of TBM or miliary TB following primary *M.tb* infection without the provision of BCG and TPT (6). A meta-analysis of recent studies including 137,647 TB-exposed children reported a nearly 20% risk of developing TB in young child contacts in the absence of TPT (disease spectrum not reported) (13). In high TB and HIV burden settings, the population pyramid is typically broad-based, with a high proportion of young children more commonly exposed to *M.tb* (18), increasing their risk of TB and TBM (19). Despite the increased TB risk amongst children living with HIV (7, 20) and the high TBM risk amongst adults living with HIV without antiretroviral therapy (21), there is no evidence of similar associations specifically between the risk of TBM and HIV in children (22).

### Diagnosis of TBM

Early diagnosis of TBM in children is critical to reduce death and disability (9, 10). Children with TBM often present with non-specific symptoms (23) and the typical neck stiffness of meningitis is often absent during the early disease stages (9, 24). Healthcare workers must therefore maintain a high index of suspicion for TBM, especially in TB-endemic settings (25). Cerebrospinal fluid (CSF) investigation and neuroimaging is important to establish the diagnosis but despite advances in diagnosis with Xpert MTB/RIF (including Ultra) (26, 27), most children with TBM are not bacteriologically confirmed (28, 29). Diagnosis is therefore mostly clinical and relies on combinations of clinical history and examination, CSF features (typically clear appearance, moderately raised white cell count, lymphocytic predominance, elevated protein level and hypoglycorrhachia) and neuroimaging demonstrating basal meningeal enhancement, infarction, hydrocephalus and/or tuberculomas.

### TBM Treatment and Outcome

TBM staging is determined by the level of consciousness and presence of neurological deficits, and is strongly associated with short and long-term outcomes (10). One large study of 548 children with TBM showed normal outcome in all fourteen children with stage 1 disease, while children with stage 3 disease had a 4.8 times higher risk of poor outcome (death and neurological sequelae) compared to those with stage 1 or 2 disease in multivariable analysis (28). Twelve months of antituberculosis therapy (2 months isoniazid, rifampicin, pyrazinamide and ethambutol followed by 10-months isoniazid and rifampicin) is currently recommended by the World Health Organization (WHO) (30). Following an updated systematic review and meta-analysis, WHO recently included a 6-month intensified regimen composed of daily isoniazid, rifampicin, pyrazinamide and ethionamide as an alternative option for the treatment of drug-susceptible TBM to the 12-month regimen (31). Neurosurgery also plays an important role in reducing disability. Outcomes depend on rapid reduction of bacillary load, control of inflammation, management of complications such as hydrocephalus and brain ischemia, supportive care and retention in care for TB treatment and beyond. Amongst 1,636 children with TBM, despite the availability of treatment, the case fatality was nearly 20% and only a third of children survived without neurological sequelae (2). The consequent lifelong neurological disability places a large economic and social burden on families, communities, and on health services.

## How Can TBM in Children Help us to Better Understand TB in Children and TB Epidemiology More Broadly?

**Figure 1:**
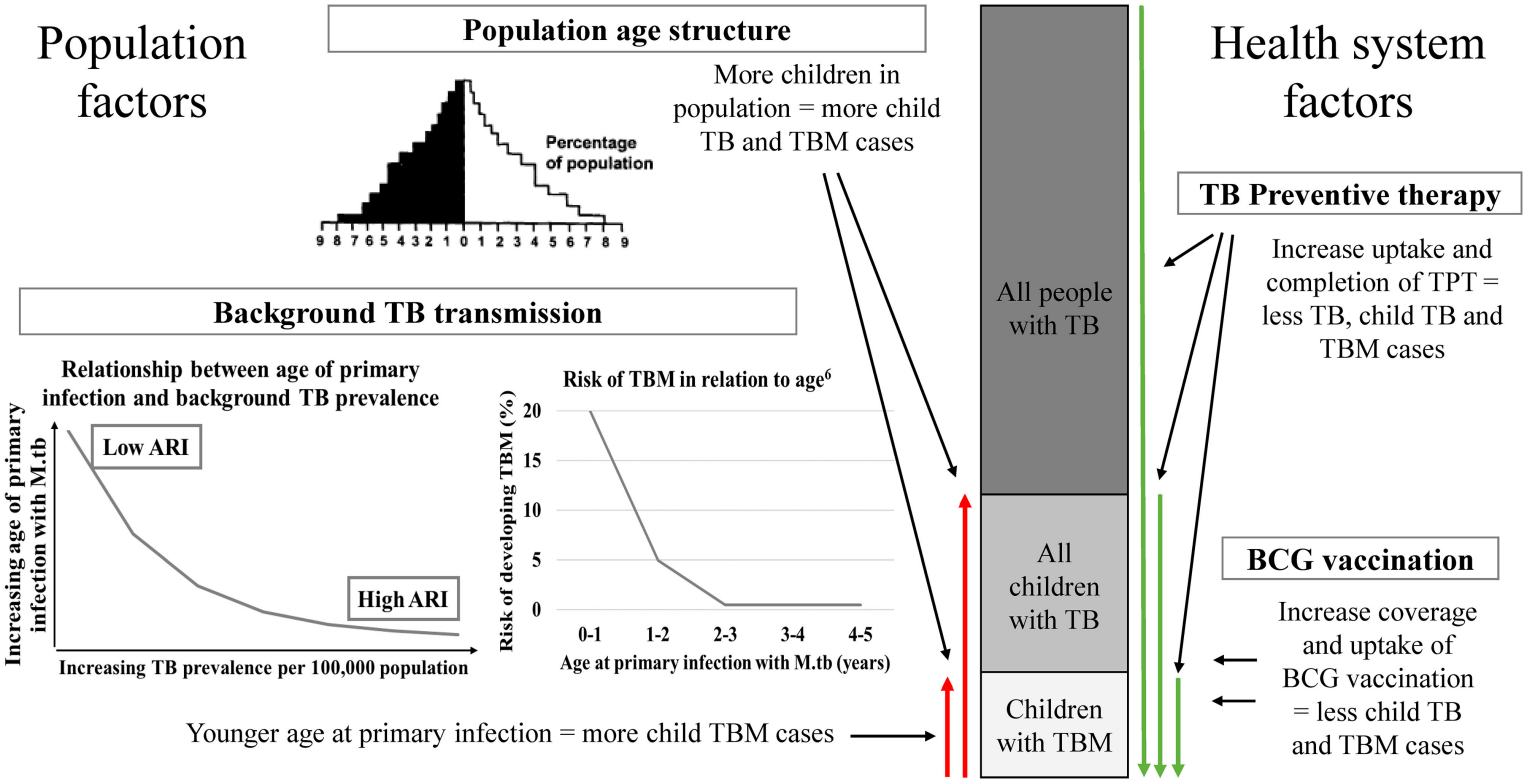
A conceptual overview of the impact of relevant population and health system factors on the relationship between pediatric TBM, pediatric TB and all TB in a population. *Population age structure*—high TB burden countries often has a broad-base population pyramid with a large percentage of the population being children. *Background TB transmission*—The higher the TB prevalence in a population, the younger the age of primary infection with *M.tb*. Following primary infection, children <2 years of age are at high risk of developing TBM (6). *TB preventive therapy* and *BCG vaccination* are two health system factors that influence the risk of TBM in children in a population. TB, tuberculosis; TBM, tuberculous meningitis; *M.tb, Mycobacterium tuberculosis*; ARI, annual risk of infection; TPT, TB preventive therapy; BCG, Bacille Calmette-Guérin.

Pediatric TBM can provide insights into the broader epidemiology of child and adult TB. Figure 1 provides an overview of population and health system factors influencing the relationship between all TB, pediatric TB, and pediatric TBM.

### Pediatric TBM as Sentinel Surveillance of the Overall TB Epidemic

Pediatric TB surveillance is an important example of sentinel surveillance of TB. A recent systematic review found that 83% of incident TB cases in children following known TB exposure were diagnosed within 90 days of their first screening visit (13). TB in young children is a sentinel epidemiological event and indicates recent transmission of *M.tb*, with surveillance providing critical insight into overall TB epidemic control.

Surveillance of pediatric TBM specifically will provide an even more sensitive marker of epidemiological control because children with TBM will either be diagnosed and treated or will die if untreated. Pre-chemotherapy era studies showed that pediatric TBM incidence was a marker for the annual risk of *M.tb* infection (ARI) and TB disease incidence overall (32–34). Data from New York City (1898–1923) showed a consistent relationship between TBM deaths and all TB deaths in children, a relationship maintained during the study period, despite a substantial fall in TB incidence (35). Another study found correlations between TBM incidence and the ARI derived from tuberculin skin test surveys: TBM incidence in children <5 years per 100,000 = ARI (%) X5 (36). The pediatric TBM caseload is therefore a robust surrogate marker of the overall TB epidemic in children, as well as of ongoing community TB transmission.

### Pediatric TBM as Marker of Health System Functioning

Surveillance of children with TBM, which are likely to present to health services rapidly, provides valuable data to identify operational health system challenges and potential solutions. Challenges include: missed opportunities for primary prevention including BCG vaccination, missed opportunities for post-exposure prevention through contact management and TPT, delayed presentation to health services, and delayed diagnosis and treatment initiation following presentation (14, 37, 38). Measuring and responding to these modifiable factors could greatly reduce the burden, morbidity and mortality of pediatric TBM and other forms of pediatric TB.

## Surveillance of Pediatric TBM: What Is the Status Quo?

Historically, TB control strategies and surveillance have focused primarily on patients contributing most to TB transmission: adults with infectious sputum smear-positive pulmonary TB (19). Young children typically develop paucibacillary TB disease, and because they contribute little to transmission, they have been largely neglected in TB control strategies and surveillance (39). In 2012, the WHO requested that countries report age-disaggregated TB data and in 2020 requested 5-year age-band disaggregated reporting for children. Despite this recent progress, substantial challenges remain for the routine surveillance of pediatric TB (40). Only 44% of the estimated number of pediatric TB cases globally were reported to WHO in 2019. Underlying challenges include under-detection of cases, incomplete reporting of detected cases, and undiagnosed deaths (40, 41).

TB programmes globally have been urged to adopt a care cascade approach for TB surveillance (42). Reporting surveillance data in a care cascade framework is a valuable tool for monitoring and evaluation of TB services by quantifying patient losses along the care cascade and informing evidence-based interventions to improve patient care. India and South Africa have both used cascade analysis to evaluate TB care (43, 44). However, these studies did not focus on children and there are limited data investigating pediatric TB using a care cascade approach. Cascade and patient pathway analysis has recently been proposed for TBM to investigate healthcare system gaps (45).

TB surveillance data should also capture the full spectrum of TB disease. However, all patients with pulmonary TB, irrespective of whether they also have extra-pulmonary TB, are currently grouped into one category when countries report disease spectrum. It is therefore impossible to distinguish TBM from other TB forms using routine TB surveillance data, and population-based estimates of the TBM burden at any age are very limited (21, 46).

In many high TB-burden countries, TB care and reporting are decentralized; hospitals do not routinely report data to TB programmes. This results in incomplete reporting of hospital-diagnosed patients, posing challenges for pediatric TB surveillance (47–50) and even more so for TBM surveillance (48). The diagnosis of TBM almost always occurs in hospital, and given the high in-hospital mortality and often in-hospital treatment completion for children with TBM, there is substantial under-reporting of pediatric TBM cases and deaths to TB programmes.

Current reporting indicators do not measure morbidity and disability post-TBM, critically important factors to comprehensively capture the pediatric TB burden. Surveillance data on delays in health care seeking, diagnosis or treatment initiation are not available and the impact of these on severity of pediatric TB and TBM disease at diagnosis is also not routinely reported.

Of key importance is that existing routine TB surveillance data are limited to only diagnosed and reported cases and do not reflect undiagnosed deaths. In high TB-burden settings, many children with TBM die undiagnosed or are misdiagnosed as having other conditions. A post-mortem study from Mozambique reported a high proportion of undiagnosed TB deaths in children at a tertiary hospital (51); In high TB-burden countries, autopsies are rare, and mortality data seldom quantify undiagnosed TB and TBM deaths accurately.

## What Has Been the Impact of COVID-19 on Pediatric TB and TBM?

The devastation of TBM has likely been compounded by the COVID-19 pandemic. Although children appear to have been spared from severe COVID-19 disease, little is known regarding the biological impact of SARS-CoV-2 on TB pathogenesis or the impact of COVID-19 public health control measures on pediatric TB services (52). Diagnosing and treating TB in children requires functional and accessible health services. If pediatric TB is diagnosed early and appropriate treatment is started, outcomes for most TB-forms are excellent (53).

WHO reported a 21% reduction in global TB case notifications between 2019 and 2020, estimating that disruptions to TB care due to COVID-19 could result in 500,000 additional TB deaths (54). The effect of COVID-19 on healthcare-seeking behavior and access to routine healthcare services is poorly understood. Reduced health seeking behavior or access to care in turn could result in children presenting with advanced TBM and could lead to more missed opportunities for TBM prevention including reduced BCG vaccination coverage (55). A recent modeling study estimated between 886 and 33,074 additional pediatric TB deaths due to COVID-19 related disruption of BCG vaccination services globally (56). Alternatively, social distancing and mask wearing to reduce COVID-19 transmission may also have reduced TB transmission, potentially lowering the rates of pediatric TB and TBM. Research to measure and understand the impact of the COVID-19 pandemic on pediatric TB and TBM is needed, especially to inform mitigation strategies for improved prevention and care of TBM in children.

## How Can We Strengthen the Surveillance of TBM in Children?

**Figure 2:**
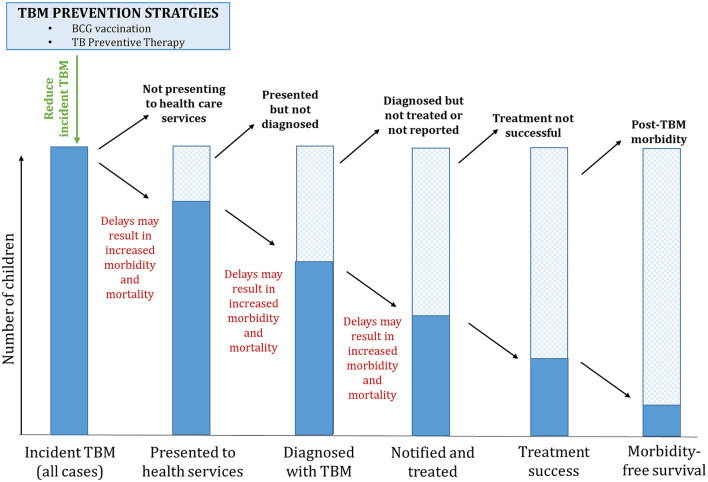
A care cascade for pediatric TBM. TBM, tuberculous meningitis; TPT, TB preventive therapy; BCG, Bacille Calmette-Guérin.

Accurate and complete pediatric TBM surveillance along the care cascade is a critical first step toward an effective response to end pediatric TB, and we therefore propose a cascade approach for children with TBM (Figure 2), including reporting of key indicators. In addition to adopting a care cascade approach, we propose the following practical recommendations to strengthen the surveillance of TBM in children (Table 1).

**Table 1:** Practical recommendations to strengthen pediatric TBM surveillance.

**Strengthen routine surveillance of children diagnosed with TBM**
• Ensure completeness of registration and reporting of all diagnosed TBM cases, including data from hospitals and both public and private sectors • Mandating the recording and capturing of all relevant ICD-10 TB diagnostic codes • Implement more comprehensive TB testing in children with meningitis living in high TB-burden countries • Ensure integration of surveillance data from multiple sources on TBM case finding and mortality
**Improve surveillance data of undiagnosed pediatric TBM deaths**
• Conduct systematic investigation of all children who die unexpectedly, especially in the presence of TB exposure and/or neurological symptoms • Undertake verbal autopsy studies and clinical reviews to identify possible misclassified deaths due to TBM
**Include pediatric TBM as key indicator in the monitoring and evaluation of pediatric TB**
• Report on pediatric TBM data along the care cascade as a routine TB programme indicator • Report on comprehensive treatment outcomes for children with TBM, including post-TBM morbidity • Report on key indicators specific to pediatric TBM prevention strategies (BCG vaccination coverage and TB contact management including TPT uptake) • Undertake operational research to identify health system challenges and solutions in the reporting of pediatric TBM

*TBM, Tuberculous meningitis; ICD, International classification of disease; TB, Tuberculosis; BCG, Bacille Calmette-Guérin; TPT, TB preventive therapy*.

### Strengthen Routine Surveillance of Children Diagnosed With TBM

Completeness of registration and reporting is essential to strengthen TBM surveillance and quantify elements of the care cascade. For example, children with TBM are mostly diagnosed in hospital. In many countries, public and/or private hospitals do not report TB surveillance data, increasing the risk of underreporting. Mandatory rapid reporting of all forms of TB in children, including TBM, as happens with “acute flaccid paralysis” surveillance to identify potential polio in children, will enhance TBM surveillance.

Surveillance systems in high TB-burden countries are moving toward electronic, individual patient records with ability to also capture and report ICD10 codes for primary and secondary TB disease sites. If diagnostic codes are completed accurately and included in reporting, it would allow more complete reporting of age-disaggregated data specifically on TBM at a global level.

Children with meningitis in high TB-burden settings could be tested for TB using samples from other clinically relevant sites in addition to CSF, such as gastric aspirates, sputum, peripheral lymph nodes, urine, blood or stool. A bacteriologically positive *M.tb* test with clinical/neuroimaging features of meningitis, could improve the level of certainty of a TBM diagnosis.

**Figure 3:**
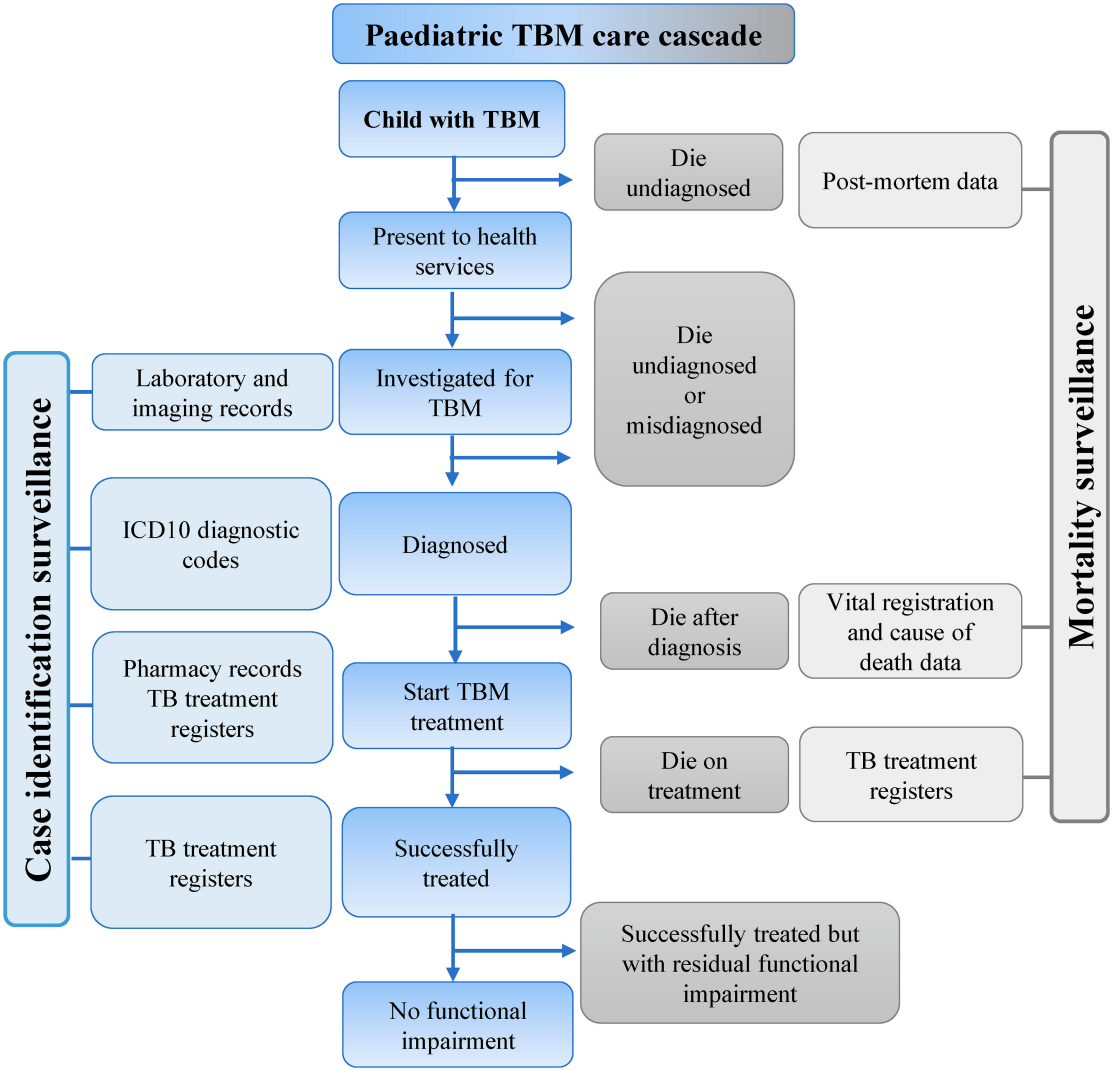
Possible sources of data to inform a pediatric TBM care cascade. TBM, Tuberculous meningitis; ICD, International classification of diseases; TB, Tuberculosis.

Integrating multiple data sources allows real-time reporting of TB surveillance data using a care cascade framework for specific sub-populations, such as children with TBM. Figure 3 provides an overview of different types of data that could be combined for these purposes. Vital registration data with accurate classification of death is essential to accurately capture TBM outcomes.

### Improve Surveillance Data of Undiagnosed Pediatric TBM Deaths

Given the high case fatality rates in the absence of treatment, pediatric TBM surveillance systems must address undiagnosed/misdiagnosed deaths. Further investigation of children with sudden unexpected deaths, especially those with presumed meningitis or a history of TB exposure, may identify undiagnosed child TBM deaths. Limited data are available on the sensitivity and specificity of TB diagnostic tests such as Xpert MTB/RIF, Xpert Ultra or *M.tb* culture on CSF at post-mortem examination, and requires further investigation.

TBM deaths might be misclassified as other conditions, such as meningitis of unknown cause, septicaemia, malnutrition or first-time status epilepticus; verbal autopsies or clinical reviews of child deaths due to these causes, specifically with a history of TB exposure, might identify incorrectly classified deaths due to TBM.

### Include Pediatric TBM as Key Indicator in the Monitoring and Evaluation of Pediatric TB

Including pediatric TBM as a TB programme indicator would require political will and support from TB programmes. Routine surveillance data should include key indicators along the pediatric TBM care cascade and in the context of overall pediatric TB care. In addition to standard TB treatment outcomes, TBM outcomes should also include post-TBM morbidity and pre-treatment mortality. BCG vaccination coverage and implementation of TPT services should be monitored as per country-specific TB guidelines. Operational research is an essential part of monitoring and evaluation and should be used alongside good quality surveillance data to identify health system challenges and solutions in pediatric TBM reporting.

## What Is the Potential Impact of Accurate Surveillance Data for Pediatric TBM?

Improved data regarding the number of children affected by TBM throughout the care cascade would have a positive impact on multiple levels (41), and is critical for advocating for the needs of children with TBM.

### Impact on Clinical Care

The lack of reliable TBM burden estimates results in inadequate resources; knowing the number of children with TBM will lead to improved resource allocation and funding toward programme implementation. Quantifying patient losses and time delays along the care cascade will in turn inform intervention planning to improve care, for example training of healthcare workers to reduce the time to referral, diagnosis and treatment. Knowledge of the number of children with TBM and how many cases are lost along the care cascade will help quantify specific problems and measure care provision for these children and their families.

### Impact on Preventive Strategies

BCG vaccination at birth and TB contact management remain the cornerstones of childhood TBM prevention strategies, and surveillance of BCG vaccination coverage and TPT uptake should be an integral part of TBM surveillance. In addition, a diagnosis of TBM can help to identify missed opportunities for prevention. Analysis of surveillance data can be used to monitor the impact of preventive strategies such as BCG vaccination coverage over time. Missed opportunities for contact management and TPT in child contacts could be quantified in order to generate momentum to strengthen child contact management and reduce the burden of TBM in children.

### Impact on Health Systems

Pediatric TBM surveillance data could assist TB programmes to monitor and evaluate pediatric TB care, identify gaps in existing health systems and inform targeted impactful public health interventions, creating urgency and mobilizing stakeholder engagement at multiple levels. Data could also inform accurate cost analysis to better motivate for adequate resources to strengthen TBM programmes and improve service provision.

## TBM in Children *Remains* a Public Health Emergency

TBM is the most devastating form of pediatric TB. It is preventable and treatable, yet still contributes substantially to child TB deaths and morbidity. A TBM diagnosis should be seen as a public health emergency and should be reported and monitored as such. Without surveillance data and a framework of reporting indicators, we will not be able to monitor trends, measure the impact of interventions, or effectively respond to this devastating form of TB in children. Surveillance data can help us identify opportunities for prevention, early diagnosis, and improved care to minimize the impact of TBM on child health globally.

## Data Availability Statement

The original contributions presented in the study are included in the article/supplementary material, further inquiries can be directed to the corresponding author/s.

## Author Contributions

KDP and JS prepared the original draft of the manuscript. All authors were involved with conceptualization of the idea, reviewed, edited the manuscript drafts, critically reviewed, and approved the final version of the manuscript.

## Funding

KDP is supported by the Fogarty International Center of the National Institutes of Health under Award Number K43TW011006. AH is financially supported by the South African National Research Foundation through a South African Research Chairs Initiative (SARChI). JS is supported by a Clinician Scientist Fellowship jointly funded by the UK Medical Research Council (MRC) and the UK Department for International Development (DFID) under the MRC/DFID Concordat agreement (MR/R007942/1). HJ is supported by the U.S. National Institutes of Health, National Institute of Allergy and Infectious Diseases (R03AI164123 and R01AI152126).

## Author Disclaimer

The content is solely the responsibility of the authors and does not necessarily represent the official views of the National Institutes of Health.

## Conflict of Interest

The authors declare that the research was conducted in the absence of any commercial or financial relationships that could be construed as a potential conflict of interest.

## Publisher's Note

All claims expressed in this article are solely those of the authors and do not necessarily represent those of their affiliated organizations, or those of the publisher, the editors and the reviewers. Any product that may be evaluated in this article, or claim that may be made by its manufacturer, is not guaranteed or endorsed by the publisher.
